# Utilising digital technologies for mycetoma management: A comprehensive review of advances and opportunities

**DOI:** 10.1371/journal.pdig.0001822

**Published:** 2026-11-06

**Authors:** Ahmad Mohammad Al Zamel, Ali Awadallah Saeed, Malaz Abdelrahman Ahmed, Samira Mohammed, Eman Kheir, Safaa Badi, Ahmed Hassan Fahal

**Affiliations:** 1 Faculty of Medicine and Surgery, Al-Neelain University, Khartoum, Sudan; 2 The Mycetoma Research Center, University of Khartoum, Khartoum, Sudan; 3 Faculty of Pharmacy, National University, Khartoum, Sudan; 4 Faculty of Medicine, University of Khartoum, Khartoum, Sudan; 5 Periodontology and Oral Medicine, Department of Periodontology, Faculty of Dental Medicine and Surgery, Centre for Professional Development, National University-Sudan, Khartoum, Sudan; 6 Department of Clinical Pharmacy, Faculty of Pharmacy, Omdurman Islamic University, Khartoum, Sudan; University of Exeter - Streatham Campus: University of Exeter, UNITED KINGDOM OF GREAT BRITAIN AND NORTHERN IRELAND

## Abstract

Mycetoma is a neglected tropical disease characterised by chronic, destructive infections of subcutaneous tissues, predominantly affecting populations in endemic regions with limited healthcare resources. Delayed diagnosis, limited treatment options, and restricted access to specialised healthcare contribute to substantial morbidity, disability, and socioeconomic burden. Digital technologies offer promising opportunities to address these challenges and improve mycetoma care. Artificial intelligence (AI) can support early detection and diagnostic accuracy through the analysis of clinical images, radiological findings, and patient data. Telemedicine can facilitate remote specialist consultations and follow-up, particularly in rural and underserved communities, while electronic health records enable systematic data collection, disease surveillance, and monitoring of treatment outcomes. Mobile health applications can improve patient education, symptom monitoring, and treatment adherence, whereas computational drug discovery may accelerate the identification of potential therapeutic agents. Furthermore, digital platforms can strengthen international research collaborations, knowledge exchange, and capacity building. However, implementing these technologies in endemic regions faces significant barriers, including inadequate digital infrastructure, limited internet connectivity, insufficient digital literacy, data privacy concerns, and ethical challenges related to informed consent, data ownership, and algorithmic decision-making. Addressing these barriers requires investment in infrastructure, appropriate regulatory frameworks, stakeholder collaboration, and context-specific capacity-building initiatives. Integrating digital health solutions into national healthcare strategies could support sustainable and equitable improvements in mycetoma management. This review highlights the potential applications, opportunities, and challenges of digital technologies in mycetoma care and emphasises the need for inclusive, locally adapted approaches to enhance early diagnosis, treatment outcomes, research, and health equity in endemic communities.

## Introduction

Mycetoma is a chronic, progressive, and often debilitating inflammatory disease that has historically received limited attention despite its significant health and socio-economic impacts [1–3]. It is caused either by certain fungi, leading to eumycetoma, or by bacteria, resulting in actinomycetoma [4–6]. The disease typically begins as a painless swelling, which gradually develops into large, disfiguring masses accompanied by multiple discharging sinuses containing characteristic grains that serve as diagnostic markers [7–9]. As the infection advances, it can invade underlying tissues, bones, and skin, causing deformities, loss of function, and in severe cases, necessitating amputation [5,10–12].

Beyond its physical effects, mycetoma considerably impacts patients’ social and psychological well-being [13]. Visible disfigurement and disability often lead to social stigma, discrimination, and mental health challenges. The disease can also lead to economic hardship, particularly for those engaged in manual labour, by causing loss of livelihoods and increasing financial strain on families and communities [14,15]. Healthcare systems in endemic areas are frequently overwhelmed by the demands of managing mycetoma, yet many lack the necessary diagnostic tools, trained healthcare personnel, and effective treatment protocols, resulting in delayed diagnoses, inadequate care, and poor outcomes [16,17].

Mycetoma predominantly affects populations residing in tropical and subtropical regions, particularly impoverished rural communities with limited access to healthcare [18–21]. Despite its substantial burden, it remains a neglected disease, receiving minimal attention from global health communities, researchers, and funding bodies [1,15,21–23]. This neglect hampers the collection of comprehensive epidemiological data, restricts research efforts, and limits the development of effective diagnostic and treatment options.

Currently, managing mycetoma remains challenging. Diagnosis relies on clinical assessment, microbiological culture, histopathology, and imaging techniques that require specialised skills and resources, often unavailable in resource-poor settings [24–26]. Treatment typically involves long courses of antifungal or antibacterial medications and surgical interventions, including amputations [27–29]. Unfortunately, these approaches often yield suboptimal results, characterised by low cure rates, high recurrence rates, and challenges with patient follow-up [29–31].

However, recent advancements in digital technology present new opportunities to revolutionise mycetoma management [32]. Innovations such as telemedicine can connect patients in remote areas with specialised healthcare providers, enabling earlier detection and consultation. Artificial intelligence (AI) and machine learning algorithms can improve diagnostic accuracy by analysing clinical, radiological, and histopathological images [32]. Mobile health (mHealth) applications have the potential to enhance patient education, promote adherence to treatment, and support follow-up care, even in areas with limited healthcare infrastructure [32]. Portable diagnostic devices and digital imaging tools facilitate early detection and ongoing disease monitoring at the community level, reducing delays in diagnosis and treatment.

Moreover, digital platforms can support epidemiological surveillance by providing valuable data on disease distribution, risk factors, and treatment outcomes. Such data can inform public health strategies, optimise resource allocation, and guide research priorities, ultimately leading to more effective control and prevention initiatives. Digital health tools also foster community engagement and awareness, helping to reduce stigma and encourage early healthcare seeking.

Despite these promising developments, integrating digital technologies into mycetoma care faces obstacles, including infrastructural limitations, shortages of trained personnel, and concerns regarding data privacy and sustainability. Building on this context, this review aims to explore the advantages and challenges of utilising technology in the management of mycetoma patients and in advancing objective scientific research.

## The use of digital technology in medical practice

In recent years, digital technology has revolutionised multiple sectors, and healthcare is no exception [33]. The integration of digital tools into medical practice has opened up new horizons for diagnosis, treatment, patient engagement, healthcare management, and scientific research [34]. This transformation is enhancing the quality, accessibility, and efficiency of healthcare delivery worldwide, ultimately leading to improved patient outcomes and more sustainable healthcare systems [35–37] (Fig 1).

**Fig 1:**
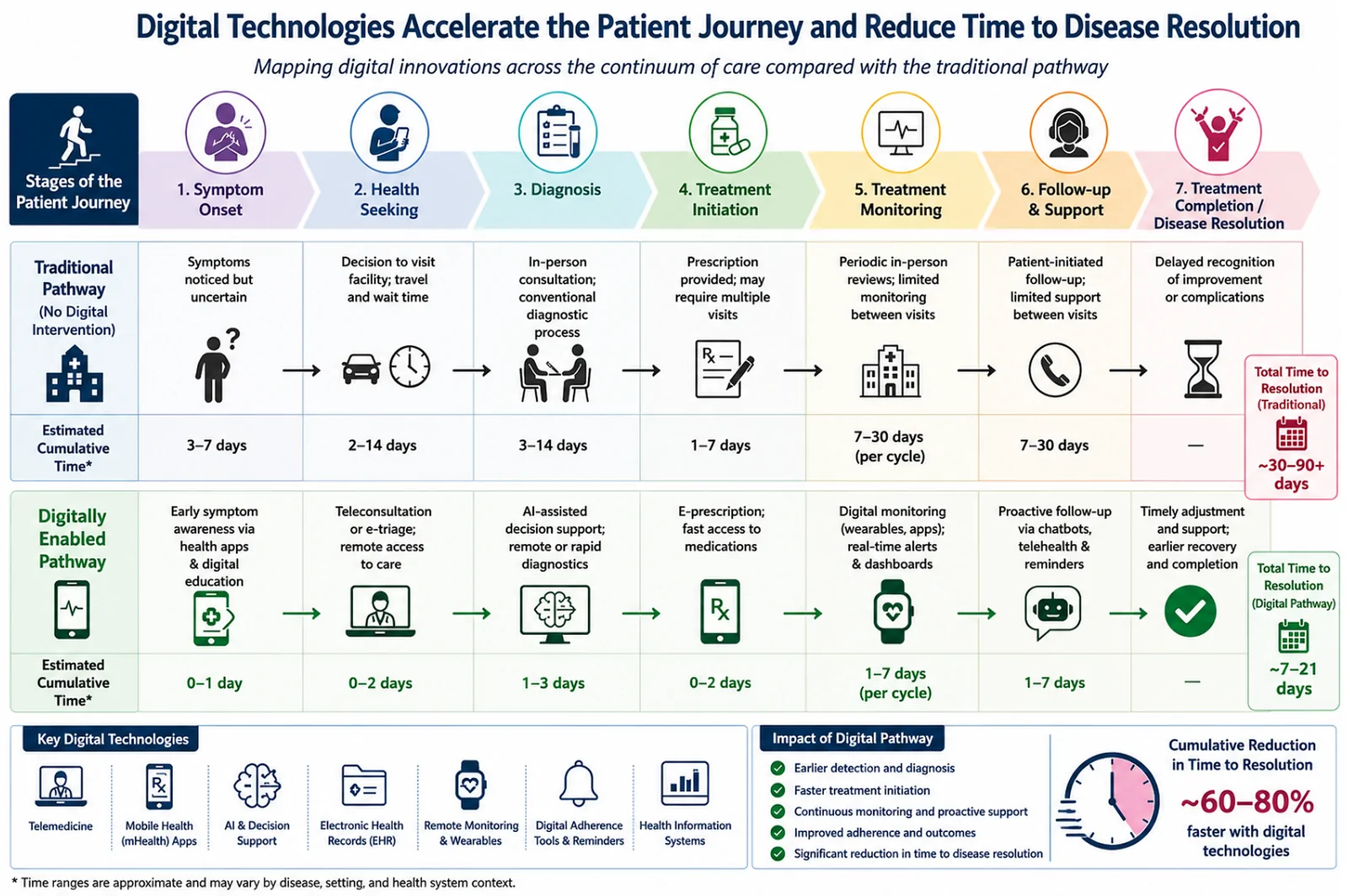
Digital Health Technologies Across the Patient Journey: From Symptom Onset to Treatment Completion: Digital health technologies, including telemedicine, mobile health (mHealth), artificial intelligence (AI), electronic health records (EHRs), geographic information systems (GIS), and digital adherence tools, enhance early detection, diagnosis, treatment, follow-up, and patient outcomes by improving access, efficiency, and continuity of care.

## Enhancing diagnosis and treatment

One of the most significant impacts of digital technology in medicine is its ability to improve diagnostic accuracy. Advanced imaging techniques, such as ultrasound, MRI, CT scans, and digital X-rays, are now complemented by artificial intelligence algorithms capable of detecting subtle patterns that may escape human detection [38,39]. AI-powered software can rapidly and precisely analyse medical images, assisting radiologists and clinicians in the early and accurate diagnosis of complex conditions, such as cancers, neurological diseases, and infectious diseases [40].

Telemedicine is another groundbreaking development, especially valuable in remote and underserved areas [41,42]. It enables healthcare providers to consult, diagnose, and monitor patients through video calls and digital platforms, reducing the need for travel and increasing access to specialist care. During the COVID-19 pandemic, telehealth services expanded exponentially, demonstrating their potential to maintain continuity of care amidst restrictions and overwhelmed healthcare facilities [43,44].

## Promoting patient engagement and self-management

Digital health tools empower patients to take an active role in their health management [45]. Mobile health (mHealth) applications enable individuals to track vital signs, medication adherence, and lifestyle factors, including diet and exercise [46]. Wearable devices, such as smartwatches, monitor heart rate, activity levels, and sleep patterns, providing real-time data that can inform healthcare decisions [47]. These technologies facilitate personalised medicine, enabling tailored interventions based on individual health metrics.

Furthermore, digital platforms offer educational resources, reminding patients about medication schedules, appointments, and lifestyle modifications [48,49]. This increased engagement can lead to improved treatment adherence, reduced hospital readmissions, and foster healthier behaviours [48].

## Improving healthcare operations and data management

Digital technology also streamlines healthcare operations through electronic health records (EHRs), which digitise patient histories, laboratory results, and treatment plans [49]. EHRs enhance coordination among healthcare providers, reduce errors, and facilitate the sharing of data across institutions. Additionally, health information systems support epidemiological surveillance, tracking disease outbreaks, and monitoring public health trends [50].

Big data analytics harnesses vast amounts of health data to identify patterns, predict disease outbreaks, and personalise treatments [51]. Cloud computing further enables secure storage and sharing of health information, fostering collaboration and research across borders [52].

## Digital technologies in mycetoma management

The integration of innovative digital technologies offers a transformative opportunity to improve the diagnosis, treatment, and overall management of mycetoma. These technologies enhance healthcare delivery by making it more accessible, efficient, and personalised, especially in underserved regions (Fig 2).

**Fig 2:**
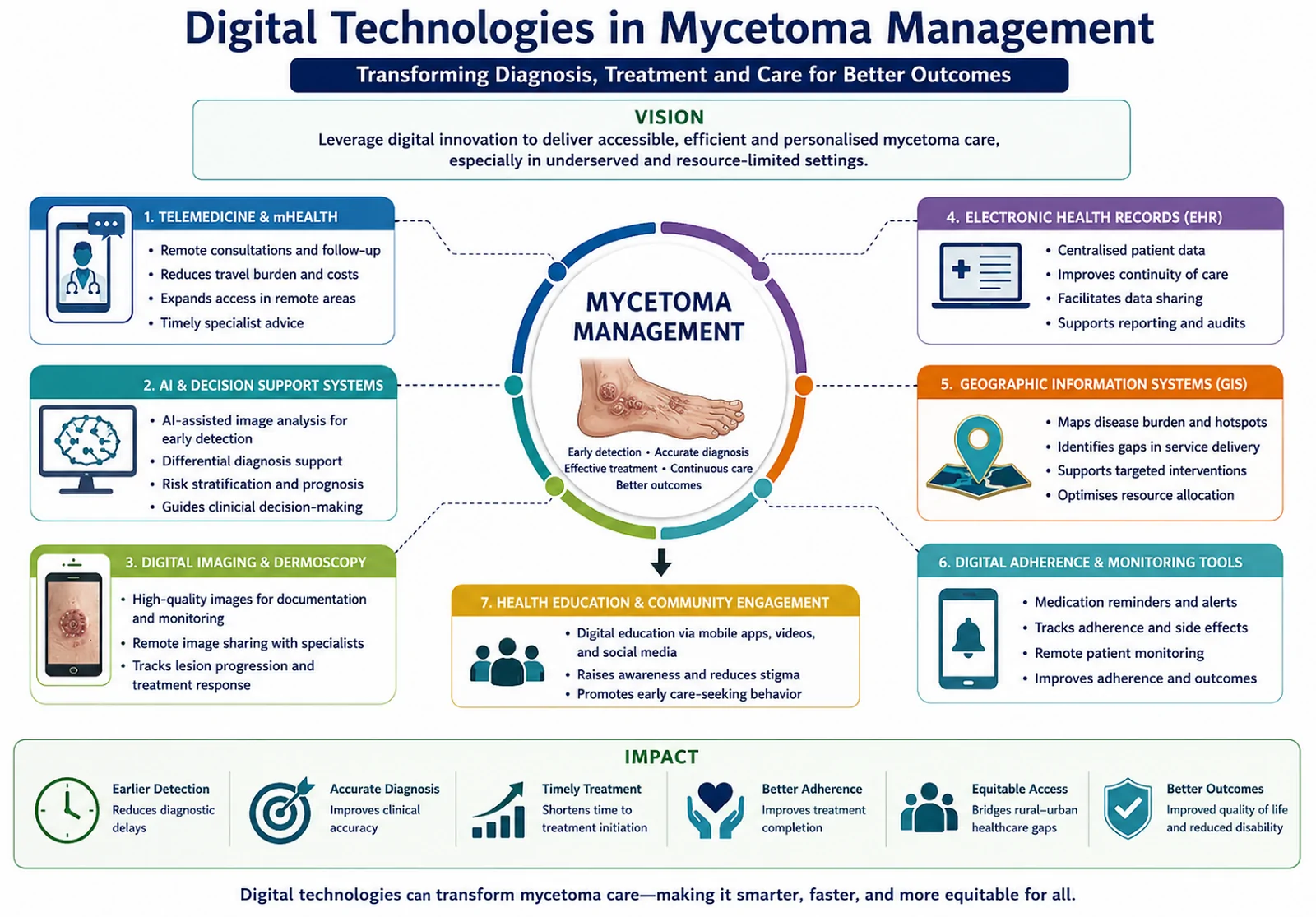
Digital Technologies in Mycetoma Management: Transforming Diagnosis, Treatment, and Patient Care: Digital technologies enhance mycetoma management by supporting early detection, accurate diagnosis, timely treatment, patient monitoring, and improved access to care, particularly in resource-limited settings.

## Improved accessibility and information dissemination

Various digital platforms enable the widespread dissemination of vital information related to mycetoma, including its clinical features, diagnostic procedures, treatment options, potential complications, and ongoing biomedical research [53–55]. This open-access information empowers both healthcare providers and patients, fostering a better understanding of the disease [58]. Such access is particularly crucial in low-resource settings, where healthcare infrastructure may be limited. By providing reliable online resources, digital technologies help bridge knowledge gaps and support informed decision-making [56,57].

The Mycetoma Research Center (MRC), University of Khartoum, is utilising digital technologies, including the effective leveraging of the Moodle e-learning platform, to deliver training modules. This involved designing and organising course materials, creating interactive content, and utilising various multimedia tools to enhance learner engagement (https://mycetoma.online/). It facilitated remote learning sessions, tracked participant progress, and provided timely feedback through the platform’s analytics and communication features. By integrating digital technologies, such as quizzes, forums, and assessments, within Moodle, a flexible, accessible, and comprehensive training environment was created that supports continuous learning and skill development for diverse audiences [58].

Patient education and community engagement are vital components of improving health outcomes, and AI chatbots play a significant role by providing personalised health information and support to encourage medication adherence and healthy behaviours. Simultaneously, social media campaigns, such as those led by the MRC (www.mycetoma.edusd), help raise awareness about various health issues and foster the development of online support networks, which can offer emotional encouragement and practical advice [59]. Together, these tools not only increase public knowledge but also help reduce the stigma associated with certain conditions, thereby encouraging individuals to seek healthcare earlier and more proactively [60,61]. This integrated approach enhances community involvement, empowers patients, and ultimately contributes to better health management at both individual and population levels.

## Telemedicine and remote consultation services

Telemedicine has emerged as a powerful tool in managing mycetoma, especially for patients residing in rural or remote areas with limited access to specialised healthcare facilities. The MRC established a telemedicine platform in 2016, connecting it with the Wad onsna Satellite Centre in Eastern Sennar Locality, Sennar State, Sudan [61]. Through this platform, healthcare professionals conduct virtual consultations, evaluate patients, interpret diagnostic images, and recommend appropriate treatment strategies without the need for physical travel. More recently, a telemedicine platform was established in collaboration with the Kassala Mycetoma Clinic, Kassala Teaching Hospital, and Kassala State. This not only accelerates diagnosis and intervention but also reduces the financial and logistical burdens on patients. Telemedicine also facilitates follow-up care, ensuring continuity and monitoring of treatment progress over time.

## Telepharmacy for mycetoma management

Online pharmacy platforms, also known as telepharmacies, play a vital role in ensuring timely access to essential medications required for mycetoma treatment. In regions where pharmacies are scarce or difficult to reach, telepharmacy services enable patients to order medicines securely and conveniently. This reduces delays in treatment initiation and adherence, which are critical factors in managing chronic infections like mycetoma. Clinical pharmacists at the MRC are in direct contact with patients through smartphones to provide medication counselling and support, enhancing patient compliance and understanding of their treatment regimens.

Moreover, the pharmaceutical industry employs digital technologies for virtual clinical trials, electronic prescriptions, e-pharmacies, and big data research [62]. These advancements optimise drug development, enhance medication accessibility, and facilitate efficient data analysis for improved decision-making in pharmaceutical care [62,63].

## Artificial Intelligence (AI) in mycetoma diagnosis and treatment

Artificial intelligence represents a rapidly expanding frontier in the biomedical sciences and healthcare [59,60]. In the context of mycetoma, AI can significantly improve diagnostic accuracy through advanced image analysis, pattern recognition, and data processing [32,64]. For example, AI algorithms were used to analyse histopathological section images to detect characteristic features of mycetoma with high precision, potentially enabling earlier diagnosis [64].

Moreover, AI can support personalised treatment planning by analysing vast datasets of patient information, treatment outcomes, and research data. This can lead to optimised therapeutic strategies, improved efficacy, and reduced side effects. AI also enhances the efficiency of biomedical research and clinical trials by automating data collection, analysis, and hypothesis generation, accelerating the development of new treatments.

## Automation and labour-intensive tasks

AI and digital automation are particularly valuable in domains requiring strenuous physical labour or repetitive tasks, such as sample processing in laboratories or administrative workflows. The MRC has a biobank comprising a vast number of microorganism isolates, DNA, histopathological tissue blocks, and environmental samples, all of which are automated. Automating these processes reduces human error, saves time, and allows healthcare providers to focus more on direct patient care. The digital solutions in laboratories enhance the management of patient data, biomarker data, logistics, and billing information. These technologies improve diagnostic precision, optimise laboratory processes, and facilitate the effective management of medical tests and specimens [65].

Digital technologies in hospitals facilitate the management of electronic health records, medical imaging data, claims processing, and billing systems [66]. These innovations enhance patient management, streamline healthcare administration, and improve financial tracking, thereby ensuring a more efficient healthcare delivery system [61].

## Mycetoma scientific research and digital technology

Research in the digital age is increasingly driven by advanced technologies that enhance understanding and innovation across various fields. In biomedical research, digital tools have revolutionised areas such as next-generation sequencing (NGS), genome-wide association studies (GWAS), and pharmacogenetics, enabling researchers to analyse vast amounts of genetic and clinical data to identify disease predispositions, drug resistance mechanisms, and personalised treatment options [67,68]. The integration of big data and collaborative research platforms enables more precise and tailored medical interventions, ultimately enhancing patient outcomes [69]. In epidemiology, artificial intelligence analyses extensive datasets, including clinical, environmental, and demographic information, to map disease distribution, identify risk factors, and inform control strategies [70]. Ecological niche modelling, combined with GIS and environmental data, has been used in Sudan to quantify mycetoma prevalence [71].

Furthermore, AI accelerates drug discovery by evaluating molecular libraries and repurposing existing pharmaceuticals. Digital platforms facilitate global collaboration among researchers, healthcare professionals, and policymakers by enabling the real-time sharing of data, knowledge exchange, and joint research efforts [72]. Cloud-based systems support the sharing of genomic data, clinical trial results, and epidemiological findings across borders [73].

## Digital technologies in mycetoma drug discovery and development

The current landscape of mycetoma treatment faces significant challenges due to the limited availability of effective therapies, especially for eumycetoma [3,27,28,74]. This underscores the critical need for innovative and accelerated approaches to discover and develop new drugs. Digital technologies are transforming the field of drug discovery by offering powerful tools that can significantly shorten development timelines, reduce costs, and enhance the precision of identifying promising therapeutic candidates [75].

Computational drug discovery methods, such as molecular docking and molecular dynamics simulations, play a critical role in understanding how potential drugs interact with specific molecular targets associated with mycetoma pathogenesis [75,76]. These tools enable researchers to virtually screen vast libraries of compounds, predicting binding affinities and the stability of drug-target interactions. By doing so, they can identify the most promising candidates for further testing, thereby reducing reliance on traditional laboratory-based screening, which is often time-consuming and costly.

Artificial Intelligence and machine learning algorithms further enhance drug discovery efforts by analysing large-scale datasets comprising chemical structures, biological activities, and existing pharmacological information [77]. AI-driven predictive modelling can rapidly evaluate thousands of chemical compounds to identify those with the highest potential to act as antifungal or antibacterial agents against mycetoma-causing organisms [78]. This approach not only accelerates the identification process but also increases the likelihood of discovering novel compounds that might have been overlooked by conventional methods [78].

Pharmacokinetic and toxicity prediction algorithms are crucial in the early phases of drug development [79]. These computational tools simulate how a drug is absorbed, distributed, metabolised, and excreted in the body, while also predicting potential toxic effects. Early assessment of these parameters helps researchers eliminate compounds with unfavourable safety profiles before investing in costly preclinical and clinical trials, thus streamlining the development pipeline and increasing efficiency [80].

Big data analytics also contribute by providing insights into the genetic, molecular, and immunological mechanisms involved in mycetoma pathogenesis. By analysing genomic data, transcriptomic profiles, and molecular pathways, researchers can identify specific targets for intervention and develop personalised treatment strategies [81]. This approach, informed by pharmacogenomics, holds the potential to tailor therapies based on an individual patient’s genetic makeup, thereby improving efficacy and reducing adverse effects.

## Remote training and capacity building

Remote training and capacity building for healthcare workers are crucial strategies to overcome the significant shortage of trained professionals in mycetoma-endemic regions, where expertise in diagnosing and managing the disease is often limited. Digital technologies offer effective solutions by providing accessible and scalable educational resources through online platforms. These include comprehensive e-learning courses, webinars, and interactive training modules that cover essential topics such as accurate diagnosis, surgical management, and pharmacological treatment options for mycetoma. AI-driven medical training tools further enhance learning by utilising virtual case studies that simulate real-world diagnostic and treatment scenarios, enabling healthcare providers to develop critical clinical decision-making skills in a safe and controlled environment. Additionally, virtual reality (VR) and augmented reality (AR) technologies can enable practical surgical training for complex procedures associated with mycetoma management, providing practitioners with a risk-free way to practice and refine their skills before performing actual surgeries [82,83]. Online knowledge-sharing platforms also play a vital role by facilitating consultation with global experts, discussing challenging cases, and staying informed about the latest research developments. Collectively, these digital initiatives equip healthcare workers with the necessary knowledge and skills, ultimately enhancing patient outcomes and bolstering healthcare systems in regions most severely affected by mycetoma.

## Reduce mycetoma burden

Early detection and diagnosis of mycetoma have been significantly improved through the use of AI-driven image analysis technologies, which enable the rapid and accurate identification of disease lesions by examining visual characteristics such as masses, grains, and discharges. These advanced computer vision models have demonstrated remarkable precision in differentiating mycetoma from other dermatological conditions, making them particularly valuable in resource-limited settings where access to specialised diagnostic tools is scarce. Furthermore, histological image analysis conducted at the MRC in Sudan has contributed to enhancing diagnostic accuracy by providing detailed insights at the tissue level [64]. When integrated with radiological imaging techniques, AI algorithms can assess the severity of the disease and help forecast treatment requirements, ensuring that patients receive timely and appropriate care. Overall, these innovative AI applications streamline the diagnostic process, reduce misdiagnosis, and support early intervention, which are critical for improving patient outcomes and managing mycetoma more effectively, especially in underserved areas.

## Electronic Health Records (EHR) for mycetoma patients

Due to the chronic and often persistent nature of mycetoma, patients require long-term follow-up to monitor disease progression, evaluate treatment outcomes, and manage potential complications. Implementing electronic health records (EHRs) significantly enhances patient care by ensuring accurate documentation, secure storage, and easy retrieval of medical information whenever needed [84].

Centralised EHR systems enable healthcare providers to maintain comprehensive records of patient histories, prescribed medications, surgical interventions, and treatment plans. This continuity of information is vital, especially when patients seek care across multiple hospitals or clinics, as it helps prevent fragmented care and ensures consistent management of their care [84].

Furthermore, integrating EHRs with mobile applications empowers patients by granting them access to their health records, medication schedules, and appointment reminders. Patients can also communicate directly with healthcare providers to report symptoms or ask questions, fostering better engagement and timely interventions [85].

Data extracted from electronic health records can serve as a valuable resource for public health officials, enabling the analysis of trends in mycetoma cases, assessing the effectiveness of current treatments, and facilitating informed resource allocation. Overall, EHRs are essential tools for enhancing clinical management, supporting public health initiatives, and advancing research in mycetoma care.

## Mobile Health (mHealth) and wearable devices for patient monitoring

Mobile health technologies, often referred to as mHealth, are revolutionising the way patients manage their health conditions and how healthcare professionals remotely monitor patient progress [86]. These innovative tools utilise smartphones, wearable sensors, and other digital platforms to provide real-time data, support adherence to treatment plans, and enhance overall health outcomes [85].

mHealth solutions empower patients to take a more active role in their healthcare by providing accessible educational resources, medication reminders, and symptom tracking tools. Smartphone applications can deliver tailored information about wound care, hygiene practices, and the importance of adhering to prescribed antifungal or antibiotic treatments [87]. This is especially critical in remote or underserved regions where access to healthcare providers may be limited, helping bridge the gap in healthcare disparities.

One of the most effective features of mHealth is the use of SMS reminders [85]. These notifications can significantly boost medication adherence by alerting patients about their dosing schedules, upcoming follow-up appointments, and wound care instructions. Consistent medication adherence is vital in preventing infections, promoting proper wound healing, and avoiding complications [85].

Wearable devices, including smart sensors and health trackers, provide continuous monitoring of physiological parameters relevant to wound healing and infection detection [88]. For instance, sensors can track inflammation levels, temperature fluctuations, or changes in wound appearance. Early detection of abnormal signs, such as increased inflammation or fever, can prompt healthcare providers to act promptly, allowing for timely interventions and reducing the risk of severe infections or complications.

By collecting detailed data on a patient’s condition over time, wearable devices facilitate more informed clinical decision-making [89]. Healthcare providers can remotely assess healing progress, adjust treatments accordingly, and provide personalised advice. This approach not only improves patient outcomes but also reduces the need for frequent in-person visits, which can be especially beneficial during pandemics or in locations with limited healthcare infrastructure.

Smartphone applications can serve as comprehensive educational tools, offering videos, tips, and guidelines on wound management, hygiene, and medication use [90]. Educating patients about their condition enhances adherence, reduces anxiety, and fosters better self-care practices.

## Digital supply chain and logistics optimisation for mycetoma management

A major obstacle to delivering effective mycetoma care is the inconsistent availability of essential medicines, diagnostic tools, consumables, and surgical instruments. These supply chain disruptions can hinder timely treatment and worsen patient outcomes. Leveraging digital technologies offers powerful solutions to enhance supply chain management, increase transparency, and ensure reliable access to vital resources.

Implementing blockchain can provide a secure and transparent way to track the movement of medications from manufacturers to end-users [84]. This technology creates an immutable ledger that verifies the origin and journey of each medicine, reducing the risk of counterfeit drugs entering the supply chain. It also improves accountability among all stakeholders, ensuring that medicines reach the right places on time [91].

AI can analyse large datasets, such as patient records, prescribing trends, seasonal variations, and regional disease prevalence, to forecast future demand for medicines and supplies [92]. This predictive insight enables health systems to anticipate shortages before they occur, allowing for proactive restocking and reducing stockouts, particularly in remote or underserved areas.

Mobile technology enables clinics and pharmacies to monitor stock levels in real-time. These systems facilitate quick updates on inventory status, enable efficient ordering, and reduce wastage due to expired medicines. By empowering local health providers with instant access to supply data, mobile platforms help prevent treatment interruptions and ensure continuous patient care [92].

Combining blockchain, AI forecasting, and mobile inventory management creates a cohesive digital ecosystem [93]. This integrated approach enhances supply chain resilience, improves transparency, and ensures that essential medicines and supplies are consistently available where they are needed most, ultimately supporting better treatment outcomes for patients with mycetoma.

## Challenges and limitations of digital innovations in mycetoma management

While the advent of digital technologies offers significant potential to transform mycetoma diagnosis, treatment, and management, several challenges and limitations must be acknowledged to ensure the successful implementation of these advancements [94].

One of the primary constraints is that AI and other digital tools cannot fully replicate the nuanced judgment, clinical intuition, and empathetic understanding that healthcare professionals bring to their work [95]. These human elements are vital for accurate diagnosis, personalised patient care, and ethical decision-making. Relying solely on technology risks oversimplifying complex clinical scenarios and may overlook contextual factors that influence patient outcomes.

Infrastructure limitations pose another significant barrier, particularly in regions where mycetoma is endemic [96]. Inadequate healthcare facilities, limited internet connectivity, and a lack of reliable electricity supply can impede the deployment and effective use of digital solutions. Furthermore, digital literacy remains uneven across different communities, with some populations lacking the skills necessary to utilise new technologies effectively. The digital divide can exacerbate existing health disparities if not adequately addressed. To overcome these hurdles, the integration of digital innovations must be accompanied by comprehensive capacity-building initiatives, infrastructure development, and supportive policy frameworks. Training healthcare workers in digital tools, improving internet access, and ensuring equitable distribution of resources are critical steps toward widespread adoption.

Regulatory challenges and infrastructural deficiencies, especially in endemic regions, further complicate the integration process. Tailored policies that address local needs and resource constraints are necessary to facilitate the responsible and sustainable use of digital tools.

## Ethical considerations in digital health

While digital technologies offer significant benefits in healthcare, their integration presents several ethical challenges. Concerns about data privacy and security raise the risk of sensitive health information breaches. Disparities in digital literacy can deepen health inequities by leaving vulnerable populations behind. Additional issues include the potential for over-reliance on technology, diagnostic errors from AI tools, and complex questions about data ownership and informed consent. To ensure responsible use, it is essential to establish clear regulations, protect patient confidentiality, and promote equitable access to healthcare. Effective deployment in mycetoma management, for example, requires careful navigation of these ethical and privacy considerations, along with collaboration among clinicians, policymakers, and community stakeholders to develop appropriate standards and frameworks.

## Looking ahead

The future of digital technology in medicine is promising. Emerging innovations, including wearable biosensors, augmented reality, virtual reality, and blockchain, are poised to transform the healthcare industry further. Personalised medicine, driven by genomics and AI, will enable highly targeted therapies. Telemedicine will become more integrated with home-based monitoring devices, resulting in seamless, patient-centred care ecosystems.

In summary, digital technology has the transformative potential to improve medicine by increasing diagnostic precision, empowering patients, streamlining healthcare processes, and enabling global collaboration. Successfully addressing current challenges is essential to ensure these innovations benefit all, making healthcare more accessible, efficient, and effective for future generations.

In the context of mycetoma, digital solutions such as AI-based diagnostics, electronic health records, mobile health (mHealth), telemedicine, virtual training, and international research collaborations can address longstanding issues in diagnosis, treatment, and healthcare access. These innovations can enhance patient care, reduce the disease burden, and support efforts to eradicate this neglected tropical disease.

Ultimately, integrating digital technologies can shift mycetoma management from a reactive to a proactive and preventive approach, leading to improved patient outcomes and reduced socio-economic impact. Achieving this vision requires coordinated efforts, strategic investments, and supportive policies to overcome existing barriers and fully realise the benefits of digital health.

## Abbreviations

AI: Artificial intelligence
AR: augmented reality
EHRs: electronic health records
GWAS: genome-wide association studies
mHealth: Mobile health
MRC: Mycetoma Research Center
NGS: next-generation sequencing
VR: virtual reality
